# Dual role mechanisms of regulated cell death in apical periodontitis: from pathogenic destruction to therapeutic potential

**DOI:** 10.1038/s41420-025-02686-4

**Published:** 2025-08-15

**Authors:** Yu Cao, Shipeng Yang, Quzhen Baima, Yuqi Zhen, Xinyue Hang, Xiuping Meng

**Affiliations:** 1 Department of Endodontics, Hospital of Stomatology, Jilin University, Changchun, Jilin PR China; 2 Department of Oral, Plastic and Aesthetic Surgery, Hospital of Stomatology, Jilin University, Changchun, Jilin PR China

**Keywords:** Cell death, Microbiology

## Abstract

Apical periodontitis (AP), a highly prevalent infectious disease driven by pathogenic microorganisms residing in periapical tissues, orchestrates a dynamic interplay between microbial virulence and host immune defenses. Emerging evidence indicates that these pathogens critically manipulate regulated cell death (RCD) pathways to subvert immune surveillance and dictate periapical bone remodeling outcomes. While RCD has traditionally been viewed as a dichotomy between pro-inflammatory destruction and anti-inflammatory repair, recent advances reveal its context-dependent duality, shaped by microbial-immune crosstalk. Despite growing interest in this field, current literature lacks a comprehensive synthesis delineating the dual-pathological impact of RCD mechanisms in AP progression, particularly their beneficial versus detrimental roles. This review critically evaluates the molecular mechanisms of RCD and crosstalk among its forms, delineating its dual roles in immune defense versus bone destruction during AP progression. We synthesize current understanding of RCD pathways in AP pathogenesis and explore therapeutically targeting these mechanisms to modulate disease outcomes. Furthermore, we explore the feasibility of developing therapeutic strategies for AP based on RCD targets and propose novel research directions to advance understanding and treatment of this condition.

## Facts

- Pathogenic microbes evade host immune defense by hijacking RCD pathways.
- Host immune cells eliminate microbial invaders and facilitate tissue repair through RCD modalities.
- Pharmacological modulation of RCD alters periapical lesion progression, offering therapeutic potential to shift the “destructive-repair” imbalance in apical periodontitis.

## Open questions

- How do pathogenic microorganisms in periapical tissues specifically modulate distinct RCD pathways to evade immune surveillance and promote chronic infection?
- Under what conditions do RCD pathways transition from protective to destructive roles in AP progression, and how is this balance regulated?
- Can therapeutic modulation of RCD crosstalk (e.g., inhibiting detrimental pathways while sparing beneficial ones) mitigate bone destruction without compromising bacterial clearance in AP?

## Introduction

Apical periodontitis (AP) is an inflammatory lesion predominantly triggered by endodontic pathogens colonizing the root canal system, featured by inflammation and destruction of periapical tissues resulting from the interaction between microbial factors and host immune response [1, 2]. When the stimulus persists, a dynamic balance is reached between pathogenic microorganisms and the host’s defense mechanisms, leading the disease into a stable, chronic state. Despite thorough root canal therapy, a classic and effective therapeutic method, secondary endodontic infections have been documented to occur in up to 39% of cases [3]. Notably, the chronic inflammatory milieu in AP involves complex cellular dynamics, including critical cell death processes that influence disease progression and resolution. Cell death is a fundamental biological process that results in the permanent cessation of all cellular functions and activities. It not only facilitates the growth, development, and maintenance of multicellular organisms by eliminating damaged or senescent cells but also mitigates the spread of pathogens by removing infected cells [4]. From a pathophysiological perspective, mammalian cell death can be conceptually stratified into two mechanistic paradigms: accidental cell death (ACD) and regulated cell death (RCD) [5]. ACD represents an uncontrolled biophysical collapse resulting from exogenic physical insults or cytotoxic chemical exposures, often triggering an inflammatory response [6]. In contrast, RCD is a genetically controlled and orderly process of cellular demise and plays a crucial role in maintaining homeostasis, tissue renewal, and organismal development. Programmed cell death (PCD) is considered a variant of RCD that occurs under physiological conditions [5, 7, 8].

Elucidating the interplay between RCD pathways and AP pathogenesis is thus critical for therapeutic innovation. Targeted modulation of specific cell death mechanisms may resolve refractory infections or mitigate detrimental inflammation [9]. Further research into RCD’s actions in AP will advance fundamental understanding and yield novel strategies for clinical intervention.

## Historical expansion of regulated cell death modalities

**Fig. 1:**
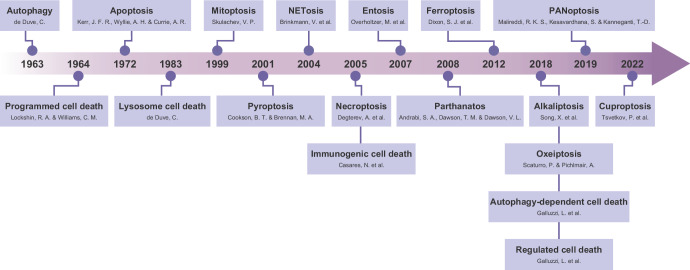
Timeline of the terms used in cell death research. Terms are displayed with their first proposed year. References for each term are listed below: Autophagy (1963) [18], Programmed cell death (1964) [11], Apoptosis (1972) [12], Lysosome cell death (1983) [21], Mitoptosis (1999) [22], Pyroptosis (2001) [16, 17], NETosis (2004) [23], Necroptosis (2005) [15], Immunogenic cell death (2005) [24], Entosis (2007) [25], Parthanatos (2008) [26], Ferroptosis (2012) [19], Autophagy-dependent cell death (2018) [5], Alkaliptosis (2018) [27], Oxeiptosis (2018) [28], Regulated cell death (2018) [5], PANoptosis (2019) [29], Cuproptosis (2022) [20].

The conceptual evolution of RCD began with Carl Vogt’s 1842 observation of physiological cell death during toad metamorphosis, where notochord removal facilitated vertebrate development [10]. The term “programmed cell death” (PCD) emerged in 1964, emphasizing genetic control over developmental cell demise [11]. John Kerr’s 1972 introduction of “apoptosis” established the first molecularly defined RCD subtype, characterized by controlled cellular deletion [12]. For decades, apoptosis dominated RCD research as its primary recognized form [13]. Necroptosis—mechanistically distinct from apoptosis—was described in 1988 but formally named in 2005 when Degterev identified its specific inhibitor Necrostatin-1 [14, 15]. Pyroptosis, an inflammatory antimicrobial defense mediated by inflammasomes, was termed in 2001 based on its proinflammatory nature [16, 17]. Autophagy first histologically noted in the nineteenth century, was molecularly defined by Christian de Duve in 1963 through lysosomal degradation studies [18]. The term “autophagy-dependent cell death” (ADCD) was formally introduced in 2018 to describe cell demise strictly requiring autophagic machinery [5]. Metabolism-associated RCD forms expanded the field, notably including ferroptosis (described in 2012) [19] and cuproptosis (described in 2022) [20]. Research spanning decades has cataloged diverse RCD modalities, such as lysosome cell death (1983) [21], mitoptosis (1999) [22], NETosis (2004) [23], immunogenic cell death (2005) [24], entosis (2007) [25], parthanatos (2008) [26], alkaliptosis (2018) [27], oxeiptosis (2018) [28] and PANoptosis (2019) [29], collectively enriching the mechanistic lexicon of RCD (Fig. 1).

## Molecular mechanisms of regulated cell death

### Apoptosis

**Fig. 2:**
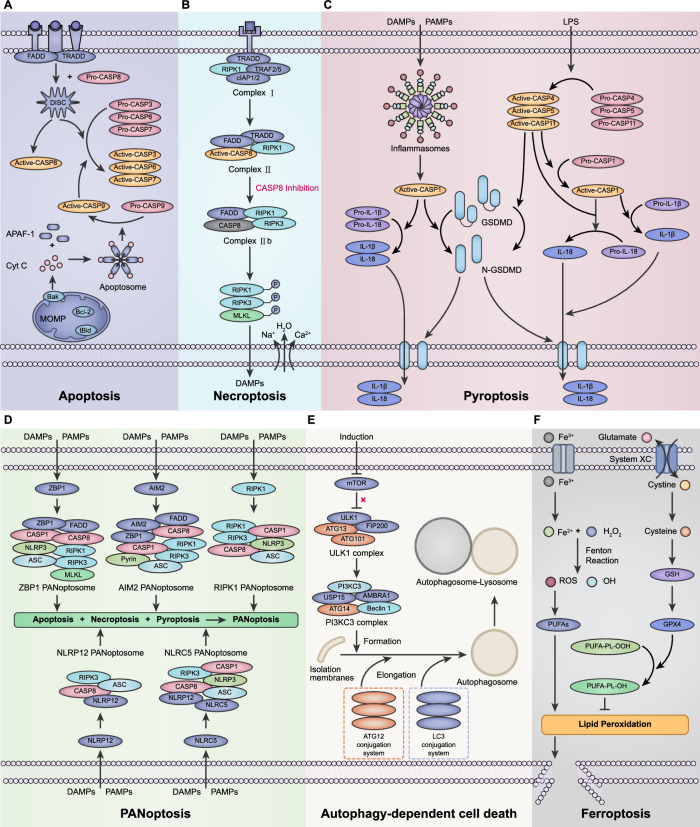
Molecular mechanisms of regulated cell death. **A** Apoptosis: intrinsic apoptosis (mitochondrial apoptosis pathway) and extrinsic apoptosis (death receptor pathway); **B** necroptosis; **C** pyroptosis: canonical pathway and non-canonical pathway; **D** PANoptosis; **E** autophagy-dependent cell death; **F** ferroptosis. Detailed molecular annotations for all signaling components are provided in Supplementary Table 1.

Apoptosis is a highly conserved form of RCD, characterized by distinct morphological and biochemical features including cell shrinkage, nuclear condensation, chromatin condensation and margination, and the formation of apoptotic bodies, while maintaining plasma membrane integrity [12]. Based on the distinct origins of apoptotic stimuli, this programmed cell death modality is categorized into intrinsic apoptosis (mediated by mitochondrial signals) and extrinsic apoptosis (regulated through death receptor-mediated processes) [30] (Fig. 2A).

Mitochondrial outer membrane permeabilization (MOMP) serves as the pivotal molecular event that governs intrinsic apoptosis regulation and is primarily mediated through dynamic interactions within the BCL-2 protein family. This protein network comprises three functional subgroups: anti-apoptotic members (Bcl-2 and Bcl-xL), pro-apoptotic effectors (Bax and Bak), and BH3-only sensitizers (Bim and Bid). Cellular stress conditions, including genotoxic damage, oxidative stress, and survival signal withdrawal, trigger the proteolytic activation of Bid to its truncated form (tBid). Activated tBid subsequently neutralizes Bcl-2/Bcl-xL mediated anti-apoptotic functions, thereby relieving the inhibition of Bax/Bak activation. This regulatory shift enables Bax and Bak to undergo conformational changes, form oligomeric complexes, and translocate to mitochondrial membranes [31–33]. Upon MOMP, cytochrome-c (Cyt C) is released into the cytoplasm, where it assembles with apoptotic protease-activating factor-1 (APAF1) to catalyze apoptosome complex formation. This complex subsequently activates caspase-9, initiating a cascade of activated caspases that irreversibly commit the cell to apoptotic death [34].

Extrinsic apoptosis, triggered by perturbations in the extracellular microenvironment, is initiated through the interaction of extracellular ligands with their cognate death receptors located on the cell membrane surface, such as TNF-αwith TNFR1, TRAIL with DR4 or DR5 (TRAILR1 or TRAILR2) and FAS-L (CD95-L) with Fas (also known as CD95 or APO-1) [5]. The binding of extracellular ligands such as TRAIL/FAS-L to their corresponding death receptors triggers receptor trimerization through structural conformational changes, exposing intracellular death domains (DDs). These conserved protein interaction modules recruit the adapter protein Fas-associated death domain (FADD) via homologous DD interactions, establishing a supramolecular platform for procaspase-8 recruitment through death effector domain (DED) homotypic binding, ultimately forming a death-inducing signaling complex (DISC) [35–37]. Then, the DISC promotes caspase-8 dimerization, facilitates the enzymatic processing of pro-caspase-8 through proteolytic cleavage, and initiates the activation of downstream effector caspases such as caspase-3, -6 and -7 [38]. Contrastingly, TNF binding to TNFR1 preferentially induces canonical pro-survival NF-κB signaling [39].

### Necroptosis

Necroptosis, a form of RCD, is activated in response to perturbations in cellular homeostasis, both extracellular and intracellular, and is detected by specific death receptors, such as Fas and TNFR [40, 41], or pathogen recognition receptors (PRRs) including Toll-like receptors (TLRs) [42]. Compared with necrosis in ACD, necroptosis shares a similar spectrum of morphological features, including cellular swelling and plasma membrane disruption, culminating in the release of intracellular components and subsequent inflammatory responses [6] (Fig. 2B).

The classical signaling pathway through which TNF activates necroptosis has been well elucidated. Under homeostatic conditions, cellular stress signals trigger caspase-8 activation to induce apoptotic pathways, whereas cells with compromised caspase activity shift to undergo necroptosis rather than apoptosis [43]. When stimulated, TNF-α links to TNFR1, which recruits TNF receptor 1-associated death domain protein (TRADD), Receptor-interacting protein kinase 1 (RIPK1), TRAF2/5, and inhibitor of apoptosis protein 1/2 (cIAP1/2) to form complex I that can be transformed to complex II [44]. Under caspase-8 suppression, RIPK1 and Receptor-interacting protein kinase 3 (RIPK3) undergo activation and mutual interaction to assemble the necroptosis-inducing complex IIb (also termed the necrosome) [45]. Subsequently, RIPK3 undergoes autophosphorylation, recruits MLKL for its phosphorylation, and thereby triggers necroptotic cell death [46]. Phosphorylated MLKL translocates to the plasma membrane, inducing Ca²⁺/Na⁺ ion influx and osmotic water entry, while triggering the release of danger-associated molecular patterns (DAMPs), ultimately culminating in the activation of pro-inflammatory signaling cascades [47–50]. Necroptosis participates in host-pathogen interactions through distinct PRR-mediated pathways. TLRs trigger necroptosis in a RIPK1 kinase activity-independent way, for example, TLR3 primarily engages double-stranded RNA (dsRNA), whereas TLR4 recognizes bacterial lipopolysaccharide (LPS), mediating TRIF recruitment to assemble the TRIF/RIPK3 signaling complex. This complex facilitates RIPK3 phosphorylation, enabling MLKL recruitment and activation to execute necroptosis [51]. Additionally, ZBP1 is activated by multiple viral pathogens through a mechanism dependent on RIPK3 recruitment but independent of RIPK1 involvement [52].

### Pyroptosis

Pyroptosis is activated by inflammasomes and executed by the gasdermin family of proteins (GSDMs) [53]. From a morphological perspective, pyroptosis presents traits similar to apoptosis, such as DNA damage at a lower intensity and chromatin condensation, but the nucleus remains intact [16], similar to necrosis, such as cell swelling, membrane permeabilization, and the release of cellular contents [54]. It is initiated through canonical and noncanonical activation of inflammasomes (Fig. 2C).

Canonical pathway (well known as caspase-1-dependent pathway) operates through inflammasome assembly, which coordinates the proteolytic cleavage of GSDMD and facilitates the secretion of mature IL-1β and IL-18. Inflammasomes, heterologous oligomeric protein complexes, constitute a robust defense mechanism against pathogens or cellular stress, leading to the execution of pyroptosis and preventing the spread of microbes while simultaneously alerting the immune system to imminent danger [55–57]. They are usually composed of three major parts: 1) Receptor proteins: PRRs (functionally categorized as inflammasome sensor components) detect PAMPs (pathogen-associated molecular patterns) and DAMPs, mainly including leucine-rich repeat containing proteins (NOD-like receptors, NLRs) and PYHIN family (HIN-200 or IFI-200family) [58, 59]; 2) Adapter protein: apoptosis-associated speck-like protein(ASC) is structurally composed of two principal functional domains: the N-terminal PYD (which interacts with certain NLR family members) and the C-terminal CARD (which interacts with pro-caspase-1) [60]; 3) Effector protein precursor: Pro-caspase-1 [61]. Following inflammasome activation, caspase-1 undergoes proteolytic maturation. Mechanistically, caspase-1 executes two parallel functions: (1) it processes gasdermin D (GSDMD) to generate an N-terminal fragment that oligomerizes into plasma membrane-perforating pores [62–64], (2) it catalyzes the maturation of pro-interleukin-1β (pro-IL-1β) and pro-interleukin-18 (pro-IL-18) into bioactive cytokines. These processed cytokines are secreted through these membrane channels, mediating immune cell recruitment to inflammatory foci and propagation of inflammatory cascades [65].

Non-canonical pathway operates through the proteolytic activation of caspase-4/5/11. These caspases can be activated by directly binding to LPS [66]. Activated caspases selectively cleave GSDMD at conserved aspartate residues, releasing its pore-forming N-terminal domain to drive osmotic lysis, which leads to cell membrane perforation, rupture, and the subsequent release of intracellular contents, thereby triggering an inflammatory response [67]. Simultaneously, they also induce the activation of caspase-1, which processes the precursors of IL-1β and IL-18, forming active IL-1β and IL-18 that are released outside the cell [68]. Moreover, Shao Feng and colleagues rigorously confirmed through in vitro and vivo experiments that caspase-4/5, activated by LPS, directly and functionally processes pro-IL-18 without inducing the maturation of IL-1β [69]. Of note, murine caspase-11, structurally and functionally similar to human caspase-4/5, is involved in the non-canonical pyroptosis pathway in mice. Research has demonstrated that the maturation of GSDMD by caspase-11 triggers two distinct cell-intrinsic signals: (1) the induction of pyroptosis; (2) the activation of caspase-1 in an NLRP3-dependent manner, thereby indirectly leading to the maturation of IL-1β and IL-18 [69, 70].

### PANoptosis

PANoptosis constitutes a synergistic inflammatory cell death modality that amalgamates core features from apoptosis, necroptosis, and pyroptosis, yet defies categorization under any singular cell death pathway [71] (Fig. 2D).

The initiation of PANoptosis is contingent upon the recognition of PAMPs or DAMPs by PRRs, including ZBP1, AIM2, RIPK1, NLRP12 and NLRC5 [72–76]. These sensors are capable of identifying specific pathogen components or intracellular stress signals, thereby triggering the assembly process of the PANoptosome. Upon activation, these sensors recruit adapter molecules, including ASC and FADD, which facilitate the transduction of signals to downstream effector molecules. Within the PANoptosome, effector molecules such as RIPK1, RIPK3, caspase-1, and caspase-8 are activated [77]. These molecules assemble into the PANoptosome complex through their respective structural domains and interaction motifs. This complex serves as a molecular scaffold, permitting the coupling and binding of key molecules involved in pyroptosis, apoptosis, and necroptosis [78].

### Autophagy-dependent cell death (ADCD)

Autophagy-dependent cell death (ADCD) has been defined as a form of RCD that mechanistically depends on the autophagic machinery (or components thereof), which is a significant distinction given that autophagy can also coincide with other forms of cell death [5]. The most studied form of autophagy is macroautophagy, henceforth referred to simply as autophagy. Autophagy is an essential, homeostatic process by which cells breakdown their own components and its morphological feature is the observation of double-membrane phagocytic components containing vesicles, namely, autophagosomes [79] (Fig. 2E).

The process of autophagy can be divided into at least four discrete steps: induction, formation of the autophagosome, autophagosome docking and fusion with the lysosome or vacuole, and breakdown of the autophagic body [80]. Autophagy induction is typically triggered by conditions such as nutrient deficiency, microbial invasion, hypoxia, or endoplasmic reticulum stress, mediated through suppression of the mechanistic target of rapamycin (mTOR). During autophagosome formation, molecular regulation occurs through sequential complexes. Nucleation requires the Unc-51-like kinase 1 (ULK1) complex (comprising ULK1, ATG13, FIP200, and ATG101) and the class III phosphatidylinositol-3-OH kinase (PI3KC3) complex (containing VPS34, VPS15, AMBRA1, Beclin 1, and ATG14). ULK1 complex activation initiates PI3KC3 assembly, catalyzing phosphatidylinositol-3-phosphate (PI3P) synthesis to recruit effectors for isolation membrane biogenesis. Subsequent elongation involves the ATG12 conjugation system (ATG12-ATG5-ATG16L) and LC3 conjugation system (PE-conjugated LC3), with phagophore membrane closure requiring additional ATG proteins [81–83]. Autophagosome-lysosome fusion depends on SNARE proteins and is promoted by tethering factors [84]. Final breakdown of the autophagic body occurs through lysosomal acidic hydrolases that degrade cargo, with retrotransported metabolites enabling cellular recycling and metabolic repurposing [82].

With the advancement of related studies, a novel variant of ADCD, termed Autosis, is executed through the reliance on the plasma membrane Na+/K+-ATPase [85]. While our current understanding of these mechanisms is not comprehensive, it is anticipated that future research will shed light on these processes.

### Ferroptosis

Ferroptosis, an iron-dependent RCD, is governed by iron metabolism, lipid peroxidation, and antioxidant systems and plays a role in various biological processes, including infectious, inflammatory, and immune diseases [86, 87]. Morphologically, it is characterized primarily by significant changes in mitochondria, such as mitochondrial shrinkage, reduction or disappearance of cristae, increased membrane density, and outer membrane rupture, whereas changes in nuclear morphology are not apparent [88] (Fig. 2F).

Ferroptosis is triggered by two key initial signals including excessive iron accumulation and the inhibition of glutathione peroxidase 4 (GPX4) [87]. Fe^3+^ enters the cell via transferrin receptor 1 (TFR1) and is reduced to Fe^2+^ within endosomes, ultimately entering the labile iron pool in the cytoplasm [89]. Due to pathological conditions caused by infection, inflammation, and immune factors that disrupt iron homeostasis, cellular iron overload can occur [90]. Excess Fe^2+^ can react with hydrogen peroxide through the Fenton reaction, forming hydroxyl radicals and highly reactive oxygen species, which can directly react with polyunsaturated fatty acids (PUFAs) in the cell membrane and plasma membrane, further promoting the peroxidation of PUFAs and leading to cell death [91].

The cystine/glutamate antiporter system (also known as System XC−) is responsible for the exchange of extracellular cystine for intracellular glutamate, a process critical for the maintenance of intracellular cysteine and glutathione (GSH) levels [92]. Cystine, once internalized, is rapidly reduced to cysteine and utilized for the biosynthesis of GSH, a principal intrinsic antioxidative molecule that serves an essential function in mitigating oxidative damage and suppressing the lipid peroxidation cascade [93]. Glutathione peroxidase 4 (GPX4), a critical negative regulatory factor [94], enzymatically neutralizes lipid hydroperoxides generated on PUFA-containing phospholipids by utilizing reduced GSH as an essential redox cofactor, catalyzing the conversion of peroxidized PUFA-phospholipids (PUFA-PL-OOH) into redox-inert alcohols (PUFA-PL-OH). This antioxidant mechanism critically mitigates membrane lipid peroxidation, thereby blocking the initiation of ferroptosis [95]. However, when the activity of System XC− is compromised, such as by the ferroptosis inducer erastin, it leads to rapid depletion of intracellular GSH. This depletion diminishes the ability of GPX4 to catalyze the reduction of lipid peroxides, resulting in the accumulation of toxic lipid ROS and triggering ferroptosis [86, 96].

## Regulated cell death in apical periodontitis: destroy or repair?

The role of RCD in inflammatory oral diseases, particularly in AP, has gained increasing attention. The relationship between distinct RCD modalities and AP manifests complexity, potentially orchestrating the interplay between microbial pathogens and host immune surveillance mechanisms. Recent studies have demonstrated that pathogenic microorganisms induce RCD across heterogeneous cellular subpopulations (Table 1).

**Table 1:** Reported apical periodontal microbiological factor in the activation of regulated cell death.

RCD	Cell models	Inducers	Key molecules		Reference
			Upregulation	Downregulation	
Apoptosis	Human osteoblast-like MG63 cells	LTA from *E. faecalis*	Bax, Caspase-3	Bcl-2	[100]
	MC3T3 osteoblasts	*E. faecalis*	Bax, Caspase-3	Bcl-2	[101]
	Human osteosarcoma MG63 cells	*E. faecalis* OG1RF	Caspase-3/-8/-9	Death receptor 6	[102]
	Human calvarial osteoblasts	*E. faecalis* OG1RF	Caspase-3/-8/-9, pro-apoptotic BCL2	anti-apoptotic BCL2	[103]
	Human periodontal ligament cells	H_2_O_2_ and lipoproteins from *S. gordonii*	–	–	[145]
Necroptosis	RAW264.7 macrophages	*E. faecalis*	RIPK3, MLKL	–	[105]
	Human osteoblast-like MG63 cells	*E. faecalis*	RIPK3, MLKL	–	[146]
	L929 cells	*F. nucleatum*	RIPK3, MLKL	–	[132]
	Oral epithelial cells	LPS from *P. gingivalis*	RIPK3, MLKL	–	[147]
	THP-1 cells	*P. gingivalis*	RIPK3, MLKL	–	[106]
Pyroptosis	THP-1 macrophages	*E. faecalis*	Caspase-1, NLRP3, GSDMD, IL-1β	–	[108]
	Human gingival fibroblasts	LPS	Caspase-1, NLRP3, IL-1β	–	[148]
	THP-1 macrophages	LPS from *P. gingivalis*	Csapase-1/-11, Caspase -4/-5, IL-1β	–	[130]
	U937 macrophages	*P. gingivalis*	NLRP3, Caspase-1/-11, GSDMD, IL-1β, IL-18		[138]
	Mouse periodontal ligament fibroblasts	LPS from *P. gingivalis*	NLRP3, Caspase-1, IL-1β	–	[149]
	Human periodontal ligament fibroblasts	LPS	NLRP3, Caspase-1	–	[107]
PANoptosis	RAW264.7 macrophages	*E. faecalis* CA1, CA2 and OG1RF	NLRP3, Caspaes-1, GSDMD Caspase-3, RIPK3, MLKL, IL-1β, IL-18	–	[99]
	RAW264.7 macrophages	*F. nucleatum*	ZBP1, GSDME, Caspase-3, MLKL	–	[114]
Autophagy	RAW264.7 macrophages	LPS	–	TFEB, LC3	[115]
	RAW264.7 macrophages	LTA from *E. faecalis*	LC3, Beclin1	–	[150]
Ferroptosis	Human gingival fibroblasts	LPS from *P. gingivalis*	TFR1, ROS	GPX4, GSH	[142]
	RAW264.7 macrophages	LPS	Fe^2+,^ ROS	GPX4, FSP1	[119]

## Destruction in apical periodontitis: promotes inflammation and bone destruction

Invasive pathogenic microorganisms, in order to survive and disseminate within periapical tissues, often silence the function of immune cells through various mechanisms, such as manipulating the apoptosis of immune cells. Evidence indicates that *Enterococcus faecalis*, which is considered the predominant causative agent of persistent AP and unsuccessful root canal treatments, modulates macrophage apoptosis in a concentration-dependent manner. At low multiplicity of infection, sustained exposure to *E. faecalis* downregulates apoptotic signaling in differentiating bone marrow stem cell-derived macrophages [97]. Additionally, early infection at high multiplicity of infection inhibits caspase-3 activation via the PI3K/Akt pathway, thereby suppressing apoptosis [98]. Conversely, high-concentration *E. faecalis* infections consistently activate caspase-dependent apoptosis in macrophages [99]. This biphasic response suggests a bacterial survival strategy: delaying host cell apoptosis during initial colonization to facilitate adaptation and spread, while later promoting apoptosis to enable bacterial release and the dissemination of periapical inflammation. Additionally, *E. faecalis* induces osteoblast apoptosis through both extrinsic and intrinsic pathways, resulting in periapical bone resorption [100–103]. Parallel mechanisms are observed in other endodontic pathogens; for example, *Fusobacterium nucleatum* isolated from human necrotic dental pulp triggers tumor necrosis factor receptor p55-mediated apoptosis in lymph node cells [104].

Necroptosis serves as a pivotal driver of inflammatory amplification and osteolytic destruction in AP, operating through regulated necrosis that releases DAMPs and proinflammatory cytokines to exacerbate periapical bone resorption. Specifically, Xing Zhu Dai et al. observed extensive macrophage infiltration in periapical lesions, with the necroptosis executioner protein MLKL co-localizing with macrophage markers CD68 and F4/80. This spatial association indicates that macrophage necroptosis directly amplifies inflammatory cascades within periapical tissues. Furthermore, pharmacological inhibition of RIPK3 (a key upstream regulator of necroptosis) significantly attenuated inflammation and reduced bone destruction in AP models [105]. Notably, MLKL suppression not only decreased TNF-α and IL-6 expression but also enhanced the clearance of *Porphyromonas gingivalis* and mitigated osteolysis, underscoring the dual role of necroptosis in both immune activation and pathological bone loss [106].

Pyroptosis, a lytic and highly pro-inflammatory form of RCD, serves as a central driver of periapical bone destruction in AP by orchestrating inflammasome-mediated cytokine release, osteoclast hyperactivation, and feedforward inflammatory amplification [107]. Mechanistically, *E. faecalis* infection triggers NLRP3 inflammasome assembly and caspase-1 activation in macrophages, inducing proteolytic maturation of IL-1β and GSDMD-mediated plasma membrane permeabilization to execute pyroptosis [108]. *P. gingivalis* activates the NLRP3 inflammasome in THP-1 macrophages independently of gingipain virulence factors [109] and also triggers IL-1β secretion and pyroptosis through co-activation of NLRP3 and AIM2 inflammasomes in the same cell type [110]. The pyroptosis-derived IL-1 critically exacerbates inflammatory osteolysis through dual pathways: (1) upregulating RANKL expression in osteoblasts to accelerate osteoclastogenesis [111], and (2) recruiting additional macrophages to amplify local inflammation [112]. Furthermore, NLRP3 inflammasome signaling inhibits regulatory T cell differentiation, thereby disrupting immune homeostasis and further promoting osteoclast-mediated bone loss [113]. Collectively, these cascades establish pyroptosis as a master regulator that integrates microbial challenge, cytokine storm, and dysregulated bone remodeling in AP pathogenesis.

PANoptosis has been shown to exacerbate inflammatory responses in periapical tissues. *F. nucleatum* infection triggers ZBP1-mediated PANoptosis in macrophages, characterized by the release of abundant inflammatory cytokines [114].

Autophagy serves as a pivotal immunomodulator in AP, dynamically modulating inflammatory responses and cellular homeostasis through context-dependent activation or suppression. Reduced transcription factor EB (TFEB)-mediated autophagy exacerbates inflammation in AP, evidenced by elevated IL-1β, IL-6, and TNF-α production in lipopolysaccharide-stimulated macrophages, whereas regulator of G protein signaling 10 overexpression activates TFEB-mediated autophagy, thereby suppressing macrophage infiltration and bone resorption [115]. Conversely, another research demonstrates enhanced autophagic activity in apical periodontitis, evidenced by concurrent upregulation of autophagy-related proteins such as beclin-1 and ATG5, increased autophagic flux, and p38 MAPK; whereas selenium inhibits autophagy activity to reduce inflammation and bone loss [116]. This finding contradicts the conventional paradigm that autophagy primarily exerts a pro-survival role in certain contexts. Although autophagy activation typically resolves inflammation by clearing damaged cells and inflammasomes [79], its inhibition-mediated anti-inflammatory effects remain mechanistically unresolved. Within the context of AP, the proliferation of intracellular pathogens deprives infected cells of oxygen, and the inflammatory response induced by bacterial infection reduces local blood flow, leading to hypoxia [117]. Elevated expression levels of hypoxia-associated proteins (HIF-1α, BNIP3, pAMPK) and autophagy-related proteins (LC3, beclin-1, Atg5-12, p62) in periapical lesion samples indicate a potential pathogenic role for hypoxia-induced autophagy in lesion progression and persistence [118].

Based on the integrated evidence, ferroptosis serves as a pivotal pathogenic mechanism in apical periodontitis by driving inflammation-triggered osteolysis through iron-dependent lipid peroxidation, macrophage-osteoblast/osteoclast dysregulation, and aberrant fatty acid metabolism. Mechanistically, ferroptotic macrophages activate a self-amplifying TNF-α autocrine-paracrine loop via NRF2/FSP1/ROS signaling, which directly suppresses the osteogenic differentiation of bone marrow stromal cells, thereby accelerating inflammatory bone destruction in apical lesions [119]. Furthermore, analogous ferroptosis activation in osteogenic-lineage cells exacerbates alveolar bone resorption and inflammation in periodontitis models, correlating with increased osteoclast formation and impaired osteogenic potential [120]. Notably, dysregulated iron and lipid metabolism—core triggers of ferroptosis—are intrinsically linked to AP pathogenesis. Atherosclerotic rats exhibit intensified periapical inflammation and bone loss due to systemic lipid dyshomeostasis [121]. Experimental murine models demonstrate that 5-lipoxygenase deficiency exacerbates the disease response by disrupting fatty acid oxidation pathways [122]. Importantly, nutritional interventions such as omega-3 polyunsaturated fatty acid supplementation attenuate pro-inflammatory cytokine infiltration and reduce osteolysis [123], whereas exogenous palmitic acid amplifies chemokine ligand 2 (CCL2) secretion and macrophage chemotaxis, exacerbating periapical tissue destruction [124]. Consequently, targeting ferroptotic signaling hubs—particularly iron homeostasis, lipid peroxidation cascades, and fatty acid metabolic checkpoints—represents a promising therapeutic strategy to mitigate inflammation-driven bone resorption and improve clinical outcomes in apical periodontitis.

## Repair in apical periodontitis: regulates inflammatory response and promotes tissue repair

Following non-surgical pulp therapy, reduced inflammatory mediators initiate apoptosis of epithelial and inflammatory cells in periapical lesions, facilitating tissue repair [125]. One research has revealed that treatment with simvastatin significantly increases the number of osteoblasts synthesizing Beclin-1, suggesting that simvastatin may alleviate the progression of AP by promoting autophagy to protect osteoblasts from apoptosis [126]. Interestingly, another study demonstrated that following hypoxic treatment, PINK1/Parkin rapidly accumulates on mitochondria, indicating a surge in mitophagic activity. Simvastatin treatment can alleviate bone resorption in AP possibly by inhibiting mitophagy-related osteoblast apoptosis [127]. In periodontal ligament stem cells from periodontitis patients, autophagic vacuoles and LC3 expression indicate autophagy’s protective role against apoptotic death [128]. While autophagy-apoptosis crosstalk requires further elucidation, accumulated evidence confirms autophagy critically facilitates periapical bone repair—particularly during osteoblast differentiation and mineralization, where its inhibition impairs mineralization and reduces bone mass [129].

The functional duality of pyroptosis in AP pathophysiology reveals stage-specific roles: at initial stages or minimal lesions, it may exert protective effects against excessive bone loss, while acute inflammation triggers significant pyroptosis exacerbating tissue damage [107, 130]. Consistent with this paradigm, ASC-knockout mice exhibit delayed osteoblast differentiation in tibial defects [131], suggesting precisely regulated pyroptosis is essential for bone repair.

**Fig. 3:**
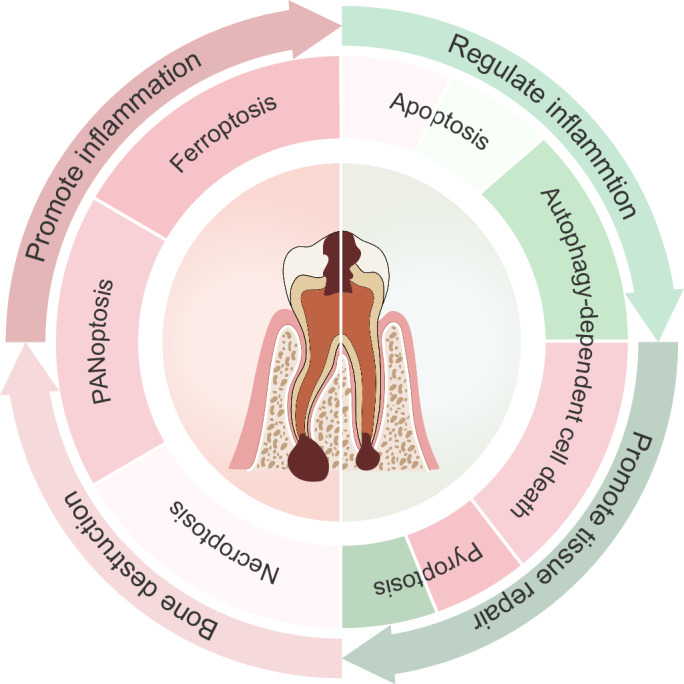
The double-edged sword effect of regulated cell death in apical periodontitis.

Current research has elucidated the double-edged sword effect of RCD in AP. To eliminate invading pathogens and maintain homeostasis, the host activates multiple signaling pathways to combat infection. However, the downstream RCD in these pathways is often associated with inflammation (such as necroptosis, pyroptosis, and PANoptosis), as its destructive effects often outweigh its reparative functions (Fig. 3). Therefore, future research should focus on elucidating the upstream signaling mechanisms by which pathogens induce RCD and explore strategies to modulate RCD in AP, aiming to optimize its reparative effects while minimizing its destructive potential, and develop new therapeutic strategies based on RCD.

## Promising therapeutic strategies based on regulated cell death for apical periodontitis

As part of their infection strategy, pathogenic microorganisms often inhibit apoptosis in host cells. *E. faecalis* can induce apoptosis in human osteoblasts and that the mRNA expression of anti-apoptotic Bcl-2 is downregulated, suggesting that BCL-2 family members may act as regulators of osteoblast apoptosis [103]. The bacterial induction of necroptosis to promote acute inflammation can lead to periapical bone destruction. RIPK3 inhibition reduces bone resorption and inflammatory cytokines in murine models [132]. Furthermore, the use of MLKL inhibitors significantly enhances the clearance of *P. gingivalis* while attenuating TNF-α and IL-6 production by 60% [106]. Similarly, Dai, X. et al. demonstrated that the use of the RIPK3 inhibitor GSK’872 or the MLKL inhibitor GW806742X can alleviate necroptosis induced by *E. faecalis* in macrophages and significantly reduce the inflammatory response [105]. Pyroptosis disrupts intracellular pathogen replication niches, enabling phagocytic clearance [133]. Nevertheless, excessive pyroptosis can intensify inflammatory responses and sustain tissue damage. Caspase-1 inhibitor VX765 mitigates bone loss and inflammation in AP [107], and NLRP3 inhibitor MCC950 reduces alveolar bone loss, IL-1β activation, and osteoclast differentiation in periodontitis models [134]. Within the NLR family, the roles of other members in pyroptosis have been investigated in detail. NLRP6 contributes to the alleviation of inflammatory responses rather than promoting pyroptosis in AP. The absence of NLRP6 leads to the upregulation of NLRP3, Pro-IL-1β, and pro-caspase-1, and it negatively regulates the production of IL-6 and TNF-α in human periodontal ligament cells by inhibiting the NF-κB and ERK signaling pathways [135]. Of note, miRNAs have recently been recognized as crucial regulators of the NLRP3 inflammasome [136]. For instance, miR-223 has been shown to suppress pyroptosis by targeting NLRP3, thereby inhibiting the activation of caspase-1 and the maturation of IL-1β [137], as does miR-155 [138]. Complementing these regulatory mechanisms, Ginsenoside Rb1 has been demonstrated to attenuate AP progression by specifically targeting calcium overload-dependent macrophage pyroptosis [139]. The Keap1/Nrf2 signaling pathway is recognized as one of the most critical cellular antioxidant systems [140], and recent research has highlighted the potential of Nrf2 activators in mitigating inflammation and tissue destruction associated with periodontal tissue. Bomidin can inhibit ferroptosis and reduce periodontal inflammation by upregulating this signaling pathway [141]. Furthermore, Ferrostatin-1 effectively inhibits P. gingivalis-LPS-induced ferroptosis, and significantly reduces the inflammatory response of human gingival fibroblasts [142]. Recent evidence demonstrates that pharmacological inhibition of the JNK/JUN/NCOA4 axis by SP600125 suppresses macrophage ferroptosis and consequently alleviates periapical inflammation [143]. Although these inhibitors show therapeutic promise by modulating inflammatory RCD pathways, their application must balance efficacy against infection spread risks. At present, root canal therapy and microsurgical apical surgery are the primary treatment methods for AP, underscoring the need for targeted RCD adjunct therapies with precise periapical delivery.

The intricate crosstalk among various forms of RCD can facilitate the shift from pro-inflammatory modes, such as necroptosis, pyroptosis, and PANoptosis, to more immunologically silent modes like apoptosis and autophagy, thereby controlling infections with reduced inflammation. Mechanistically, ubiquitin-specific protease 19 acts as an anti-inflammatory switch that inhibits NLRP3 inflammasome activation by enhancing autophagic flux and reducing mitochondrial reactive oxygen species production. This suppression of inflammatory responses concurrently promotes M2-like macrophage polarization [144]. These findings underscore the interplay between RCD-dependent macrophage polarization and crosstalk among RCD pathways.

## Conclusion

In the context of AP, the lesion is a battleground where pathogenic microorganisms are in constant struggle with the host’s immune defenses. RCD plays a multifaceted role in this conflict: eradicating pathogens, augmenting inflammatory signaling, engaging in innate and adaptive immunity, and modulating the disease trajectory. The intricate interplay between microbial pathogens and the host constitutes a sophisticated biological mechanism: pathogenic microorganisms may modulate RCD to evade the host’s immune defenses, while host immune cells can invoke various forms of RCD to clear invading pathogenic microorganisms and facilitate tissue repair. To date, the molecular mechanisms by which a limited variety of pathogenic microorganisms modulate RCD have been investigated. Furthermore, the crosstalk among various forms of RCD in AP lesions is still poorly understood, and the roles of emerging RCD mechanisms, such as ferroptosis and NETosis, in AP await further investigation. In conclusion, a deeper understanding of the complex double-edged sword role of RCD in AP is essential for improving disease management and clinical practice.

## Supplementary information

Supplementary Table 1

## References

[1] Siqueira JF, Silva WO, Romeiro K, Gominho LF, Alves FRF, Rôças IN. Apical root canal microbiome associated with primary and posttreatment apical periodontitis: a systematic review. Int Endod J. 2024;57:1043–58. 38634795 10.1111/iej.14071

[2] Visarnta S, Ratisoontorn C, Panichuttra A, Sinpitaksakul P, Chantarangsu S, Dhanuthai K. Macrophage polarization in human periapical lesions in relation to histopathological diagnosis, clinical features and lesion volume: an ex vivo study. Int Endod J. 2024;57:1829–47. 39222032 10.1111/iej.14138

[3] Tibúrcio-Machado CS, Michelon C, Zanatta FB, Gomes MS, Marin JA, Bier CA. The global prevalence of apical periodontitis: a systematic review and meta-analysis. Int Endod J. 2021;54:712–35. 33378579 10.1111/iej.13467

[4] Newton K, Strasser A, Kayagaki N, Dixit VM. Cell death. Cell. 2024;187:235–56. 38242081 10.1016/j.cell.2023.11.044

[5] Galluzzi L, Vitale I, Aaronson SA, Abrams JM, Adam D, Agostinis P, et al. Molecular mechanisms of cell death: recommendations of the Nomenclature Committee on Cell Death 2018. Cell Death Differ. 2018;25:486–541. 29362479 10.1038/s41418-017-0012-4 PMC5864239

[6] Chen Y, Li X, Yang M, Liu SB. Research progress on morphology and mechanism of programmed cell death. Cell Death Dis. 2024;15:327. 38729953 10.1038/s41419-024-06712-8 PMC11087523

[7] Galluzzi L, Vitale I, Warren S, Adjemian S, Agostinis P, Martinez AB, et al. Consensus guidelines for the definition, detection and interpretation of immunogenic cell death. J Immunother Cancer. 2020;8:e000337. 32209603 10.1136/jitc-2019-000337 PMC7064135

[8] Peng F, Liao M, Qin R, Zhu S, Peng C, Fu L, et al. Regulated cell death (RCD) in cancer: key pathways and targeted therapies. Signal Transduct Target Ther. 2022;7:286. 35963853 10.1038/s41392-022-01110-y PMC9376115

[9] Place DE, Lee S, Kanneganti TD. PANoptosis in microbial infection. Curr Opin Microbiol. 2021;59:42–9. 32829024 10.1016/j.mib.2020.07.012 PMC7438227

[10] Vogt KC. Untersuchungen über die Entwicklungsgeschichte der Geburtshelferkröte (Alytes obstetricans). Jent Gassmann. 1842.

[11] Lockshin RA, Williams CM. Programmed cell death—II. Endocrine potentiation of the breakdown of the intersegmental muscles of silkmoths. J Insect Physiol. 1964;10:643–9.

[12] Kerr JFR, Wyllie AH, Currie AR. Apoptosis: a basic biological phenomenon with wideranging implications in tissue kinetics. Br J Cancer. 1972;26:239–57. 4561027 10.1038/bjc.1972.33 PMC2008650

[13] Bertheloot D, Latz E, Franklin BS. Necroptosis, pyroptosis and apoptosis: an intricate game of cell death. Cell Mol Immunol. 2021;18:1106–21. 33785842 10.1038/s41423-020-00630-3 PMC8008022

[14] Laster SM, Wood JG, Gooding LR. Tumor necrosis factor can induce both apoptic and necrotic forms of cell lysis. J Immunol Balt Md 1950. 1988;141:2629–34. 3171180

[15] Degterev A, Huang Z, Boyce M, Li Y, Jagtap P, Mizushima N, et al. Chemical inhibitor of nonapoptotic cell death with therapeutic potential for ischemic brain injury. Nat Chem Biol. 2005;1:112–9. 16408008 10.1038/nchembio711

[16] Zychlinsky A, Prevost MC, Sansonetti PJ. Shigella flexneri induces apoptosis in infected macrophages. Nature. 1992;358:167–9. 1614548 10.1038/358167a0

[17] Cookson BT, Brennan MA. Pro-inflammatory programmed cell death. Trends Microbiol. 2001;9:113–4. 11303500 10.1016/s0966-842x(00)01936-3

[18] Mizushima N. A brief history of autophagy from cell biology to physiology and disease. Nat Cell Biol. 2018;20:521–7. 29686264 10.1038/s41556-018-0092-5

[19] Dixon SJ, Lemberg KM, Lamprecht MR, Skouta R, Zaitsev EM, Gleason CE, et al. Ferroptosis: an iron-dependent form of nonapoptotic cell death. Cell. 2012;149:1060–72. 22632970 10.1016/j.cell.2012.03.042 PMC3367386

[20] Tsvetkov P, Coy S, Petrova B, Dreishpoon M, Verma A, Abdusamad M, et al. Copper induces cell death by targeting lipoylated TCA cycle proteins. Science. 2022;375:1254–61. 35298263 10.1126/science.abf0529 PMC9273333

[21] De Duve C. Lysosomes revisited. Eur J Biochem. 1983;137:391–7. 6319122 10.1111/j.1432-1033.1983.tb07841.x

[22] Skulachev VP. Mitochondrial physiology and pathology; concepts of programmed death of organelles, cells and organisms. Mol Asp Med. 1999;20:139–84. 10.1016/s0098-2997(99)00008-4 10626278

[23] Brinkmann V, Reichard U, Goosmann C, Fauler B, Uhlemann Y, Weiss DS, et al. Neutrophil extracellular traps kill bacteria. Science. 2004;303:1532–5. 15001782 10.1126/science.1092385

[24] Casares N, Pequignot MO, Tesniere A, Ghiringhelli F, Roux S, Chaput N, et al. Caspase-dependent immunogenicity of doxorubicin-induced tumor cell death. J Exp Med. 2005;202:1691–701. 16365148 10.1084/jem.20050915 PMC2212968

[25] Overholtzer M, Mailleux AA, Mouneimne G, Normand G, Schnitt SJ, King RW, et al. A nonapoptotic cell death process, entosis, that occurs by cell-in-cell invasion. Cell. 2007;131:966–79. 18045538 10.1016/j.cell.2007.10.040

[26] Andrabi SA, Dawson TM, Dawson VL. Mitochondrial and nuclear cross talk in cell death: parthanatos. Ann N Y Acad Sci. 2008;1147:233–41. 19076445 10.1196/annals.1427.014 PMC4454457

[27] Song X, Zhu S, Xie Y, Liu J, Sun L, Zeng D, et al. JTC801 induces pH-dependent death specifically in cancer cells and slows growth of tumors in mice. Gastroenterology. 2018;154:1480–93. 29248440 10.1053/j.gastro.2017.12.004 PMC5880694

[28] Scaturro P, Pichlmair A. Oxeiptosis-a cell death pathway to mitigate damage caused by radicals. Cell Death Differ. 2018;25:1191–3. 29844568 10.1038/s41418-018-0134-3 PMC6030169

[29] Malireddi RKS, Kesavardhana S, Kanneganti TD. ZBP1 and TAK1: master regulators of NLRP3 inflammasome/pyroptosis, apoptosis, and necroptosis (PAN-optosis). Front Cell Infect Microbiol. 2019;9:406. 31850239 10.3389/fcimb.2019.00406 PMC6902032

[30] Nössing C, Ryan KM. 50 years on and still very much alive: ‘Apoptosis: a basic biological phenomenon with wide-ranging implications in tissue kinetics. Br J Cancer. 2023;128:426–31. 36369364 10.1038/s41416-022-02020-0 PMC9938139

[31] Kelekar A, Thompson CB. Bcl-2-family proteins: the role of the BH3 domain in apoptosis. Trends Cell Biol. 1998;8:324–30. 9704409 10.1016/s0962-8924(98)01321-x

[32] Czabotar PE, Lessene G, Strasser A, Adams JM. Control of apoptosis by the BCL-2 protein family: implications for physiology and therapy. Nat Rev Mol Cell Biol. 2014;15:49–63. 24355989 10.1038/nrm3722

[33] Tait SWG, Green DR. Mitochondria and cell death: outer membrane permeabilization and beyond. Nat Rev Mol Cell Biol. 2010;11:621–32. 20683470 10.1038/nrm2952

[34] Zou H, Henzel WJ, Liu X, Lutschg A, Wang X. Apaf-1, a human protein homologous to C. elegans CED-4, participates in cytochrome c-dependent activation of caspase-3. Cell. 1997;90:405–13. 9267021 10.1016/s0092-8674(00)80501-2

[35] Chinnaiyan AM, O’Rourke K, Tewari M, Dixit VM. FADD, a novel death domain-containing protein, interacts with the death domain of Fas and initiates apoptosis. Cell. 1995;81:505–12. 7538907 10.1016/0092-8674(95)90071-3

[36] Scott FL, Stec B, Pop C, Dobaczewska MK, Lee JJ, Monosov E, et al. The Fas-FADD death domain complex structure unravels signalling by receptor clustering. Nature. 2009;457:1019–22. 19118384 10.1038/nature07606 PMC2661029

[37] Lee EW, Kim JH, Ahn YH, Seo J, Ko A, Jeong M, et al. Ubiquitination and degradation of the FADD adaptor protein regulate death receptor-mediated apoptosis and necroptosis. Nat Commun. 2012;3:978. 22864571 10.1038/ncomms1981

[38] Stennicke HR, Jürgensmeier JM, Shin H, Deveraux Q, Wolf BB, Yang X, et al. Pro-caspase-3 is a major physiologic target of caspase-8*. J Biol Chem. 1998;273:27084–90. 9765224 10.1074/jbc.273.42.27084

[39] Cheng CT, Hsiao JC, Hoffmann A, Tu HL. TNFR1 mediates heterogeneity in single-cell NF-κB activation. iScience. 2024;27:109486. 38551009 10.1016/j.isci.2024.109486 PMC10973173

[40] Vercammen D, Vandenabeele P, Beyaert R, Declercq W, Fiers W. Tumour necrosis factor-induced necrosis versus anti-Fas-induced apoptosis in L929 cells. Cytokine. 1997;9:801–8. 9367540 10.1006/cyto.1997.0252

[41] Vercammen D, Brouckaert G, Denecker G, Van de Craen M, Declercq W, Fiers W, et al. Dual signaling of the Fas receptor: initiation of both apoptotic and necrotic cell death pathways. J Exp Med. 1998;188:919–30. 9730893 10.1084/jem.188.5.919 PMC2213397

[42] Takeuchi O, Akira S. Pattern recognition receptors and inflammation. Cell. 2010;140:805–20. 20303872 10.1016/j.cell.2010.01.022

[43] Fritsch M, Günther SD, Schwarzer R, Albert MC, Schorn F, Werthenbach JP, et al. Caspase-8 is the molecular switch for apoptosis, necroptosis and pyroptosis. Nature. 2019;575:683–7. 31748744 10.1038/s41586-019-1770-6

[44] Newton K. Multitasking Kinase RIPK1 regulates cell death and inflammation. Cold Spring Harb Perspect Biol. 2020;12:a036368. 31427374 10.1101/cshperspect.a036368 PMC7050590

[45] Delanghe T, Dondelinger Y, Bertrand MJM. RIPK1 kinase-dependent death: a symphony of phosphorylation events. Trends Cell Biol. 2020;30:189–200. 31959328 10.1016/j.tcb.2019.12.009

[46] Samson AL, Zhang Y, Geoghegan ND, Gavin XJ, Davies KA, Mlodzianoski MJ, et al. MLKL trafficking and accumulation at the plasma membrane control the kinetics and threshold for necroptosis. Nat Commun. 2020;11:3151. 32561730 10.1038/s41467-020-16887-1 PMC7305196

[47] Cai Z, Jitkaew S, Zhao J, Chiang HC, Choksi S, Liu J, et al. Plasma membrane translocation of trimerized MLKL protein is required for TNF-induced necroptosis. Nat Cell Biol. 2014;16:55–65. 24316671 10.1038/ncb2883 PMC8369836

[48] Chen X, Li W, Ren J, Huang D, He Wting, Song Y, et al. Translocation of mixed lineage kinase domain-like protein to plasma membrane leads to necrotic cell death. Cell Res. 2014;24:105–21. 24366341 10.1038/cr.2013.171 PMC3879712

[49] Su L, Quade B, Wang H, Sun L, Wang X, Rizo J. A plug release mechanism for membrane permeation by MLKL. Structure. 2014;22:1489–500. 25220470 10.1016/j.str.2014.07.014 PMC4192069

[50] Murai S, Yamaguchi Y, Shirasaki Y, Yamagishi M, Shindo R, Hildebrand JM, et al. A FRET biosensor for necroptosis uncovers two different modes of the release of DAMPs. Nat Commun. 2018;9:4457. 30367066 10.1038/s41467-018-06985-6 PMC6203740

[51] Kaiser WJ, Sridharan H, Huang C, Mandal P, Upton JW, Gough PJ, et al. Toll-like Receptor 3-mediated Necrosis via TRIF, RIP3, and MLKL. J Biol Chem. 2013;288:31268. 24019532 10.1074/jbc.M113.462341 PMC3829437

[52] Jiao H, Wachsmuth L, Kumari S, Schwarzer R, Lin J, Eren RO, et al. Z-nucleic acid sensing triggers ZBP1-dependent necroptosis and inflammation. Nature. 2020;580:391. 32296175 10.1038/s41586-020-2129-8 PMC7279955

[53] Yu P, Zhang X, Liu N, Tang L, Peng C, Chen X. Pyroptosis: mechanisms and diseases. Signal Transduct Target Ther. 2021;6:128. 33776057 10.1038/s41392-021-00507-5 PMC8005494

[54] Fink SL, Cookson BT. Caspase-1-dependent pore formation during pyroptosis leads to osmotic lysis of infected host macrophages. Cell Microbiol. 2006;8:1812–25. 16824040 10.1111/j.1462-5822.2006.00751.x

[55] Kesavardhana S, Malireddi RKS, Kanneganti TD. Caspases in cell death, inflammation, and pyroptosis. Annu Rev Immunol. 2020;38:567–95. 32017655 10.1146/annurev-immunol-073119-095439 PMC7190443

[56] Man SM, Karki R, Kanneganti TD. Molecular mechanisms and functions of pyroptosis, inflammatory caspases and inflammasomes in infectious diseases. Immunol Rev. 2017;277:61–75. 28462526 10.1111/imr.12534 PMC5416822

[57] Li L, Dickinson MS, Coers J, Miao EA. Pyroptosis in defense against intracellular bacteria. Semin Immunol. 2023;69:101805. 37429234 10.1016/j.smim.2023.101805 PMC10530505

[58] Amarante-Mendes GP, Adjemian S, Branco LM, Zanetti LC, Weinlich R, Bortoluci KR. Pattern recognition receptors and the host cell death molecular machinery. Front Immunol. 2018;9:2379. 30459758 10.3389/fimmu.2018.02379 PMC6232773

[59] Yao J, Sterling K, Wang Z, Zhang Y, Song W. The role of inflammasomes in human diseases and their potential as therapeutic targets. Signal Transduct Target Ther. 2024;9:10. 38177104 10.1038/s41392-023-01687-y PMC10766654

[60] Matyszewski M, Zheng W, Lueck J, Mazanek Z, Mohideen N, Lau AY, et al. Distinct axial and lateral interactions within homologous filaments dictate the signaling specificity and order of the AIM2-ASC inflammasome. Nat Commun. 2021;12:2735. 33980849 10.1038/s41467-021-23045-8 PMC8115694

[61] Martinon F, Burns K, Tschopp J. The inflammasome: a molecular platform triggering activation of inflammatory caspases and processing of proIL-beta. Mol Cell. 2002;10:417–26. 12191486 10.1016/s1097-2765(02)00599-3

[62] Liu X, Zhang Z, Ruan J, Pan Y, Magupalli VG, Wu H, et al. Inflammasome-activated gasdermin D causes pyroptosis by forming membrane pores. Nature. 2016;535:153–8. 27383986 10.1038/nature18629 PMC5539988

[63] Sborgi L, Rühl S, Mulvihill E, Pipercevic J, Heilig R, Stahlberg H, et al. GSDMD membrane pore formation constitutes the mechanism of pyroptotic cell death. EMBO J. 2016;35:1766–78. 27418190 10.15252/embj.201694696 PMC5010048

[64] Ding J, Wang K, Liu W, She Y, Sun Q, Shi J, et al. Pore-forming activity and structural autoinhibition of the gasdermin family. Nature. 2016;535:111–6. 27281216 10.1038/nature18590

[65] Lamkanfi M, Dixit VM. Mechanisms and Functions of Inflammasomes. Cell. 2014;157:1013–22. 24855941 10.1016/j.cell.2014.04.007

[66] Shi J, Zhao Y, Wang Y, Gao W, Ding J, Li P, et al. Inflammatory caspases are innate immune receptors for intracellular LPS. Nature. 2014;514:187–92. 25119034 10.1038/nature13683

[67] Aglietti RA, Estevez A, Gupta A, Ramirez MG, Liu PS, Kayagaki N, et al. GsdmD p30 elicited by caspase-11 during pyroptosis forms pores in membranes. Proc Natl Acad Sci. 2016;113:7858–63. 27339137 10.1073/pnas.1607769113 PMC4948338

[68] Shi J, Gao W, Shao F. Pyroptosis: gasdermin-mediated programmed necrotic cell death. Trends Biochem Sci. 2017;42:245–54. 27932073 10.1016/j.tibs.2016.10.004

[69] Shi X, Sun Q, Hou Y, Zeng H, Cao Y, Dong M, et al. Recognition and maturation of IL-18 by caspase-4 noncanonical inflammasome. Nature. 2023;624:442–50. 37993714 10.1038/s41586-023-06742-w

[70] Kayagaki N, Stowe IB, Lee BL, O’Rourke K, Anderson K, Warming S, et al. Caspase-11 cleaves gasdermin D for non-canonical inflammasome signalling. Nature. 2015;526:666–71. 26375259 10.1038/nature15541

[71] Zhu P, Ke ZR, Chen JX, Li SJ, Ma TL, Fan XL. Advances in mechanism and regulation of PANoptosis: Prospects in disease treatment. Front Immunol. 2023;14:1120034. 36845112 10.3389/fimmu.2023.1120034 PMC9948402

[72] Hao Y, Yang B, Yang J, Shi X, Yang X, Zhang D, et al. ZBP1: a powerful innate immune sensor and double-edged sword in host immunity. Int J Mol Sci. 2022;23:10224. 36142136 10.3390/ijms231810224 PMC9499459

[73] Lee S, Karki R, Wang Y, Nguyen LN, Kalathur RC, Kanneganti TD. AIM2 forms a complex with pyrin and ZBP1 to drive PANoptosis and host defence. Nature. 2021;597:415–9. 34471287 10.1038/s41586-021-03875-8 PMC8603942

[74] Malireddi RKS, Kesavardhana S, Karki R, Kancharana B, Burton AR, Kanneganti TD. RIPK1 distinctly regulates *Yersinia* -induced inflammatory cell death, PANoptosis. ImmunoHorizons. 2020;4:789–96. 33310881 10.4049/immunohorizons.2000097 PMC7906112

[75] Sundaram B, Pandian N, Mall R, Wang Y, Sarkar R, Kim HJ, et al. NLRP12-PANoptosome activates PANoptosis and pathology in response to heme and PAMPs. Cell. 2023;186:2783–801.e20. 37267949 10.1016/j.cell.2023.05.005 PMC10330523

[76] Jadhav PS, Mahajan S, Man SM. NLRC5 PANoptosome: Aquaman of the dead sea. Cell Res. 2025;35:9–10. 39112672 10.1038/s41422-024-01011-5 PMC11701087

[77] Pandian N, Kanneganti TD. PANoptosis: a unique inflammatory cell death modality. J Immunol Balt Md 1950. 2022;209:1625–33. 10.4049/jimmunol.2200508 PMC9586465 36253067

[78] Qi Z, Zhu L, Wang K, Wang N. PANoptosis: emerging mechanisms and disease implications. Life Sci. 2023;333:122158. 37806654 10.1016/j.lfs.2023.122158

[79] Deretic V. Autophagy in inflammation, infection, and immunometabolism. Immunity. 2021;54:437–53. 33691134 10.1016/j.immuni.2021.01.018 PMC8026106

[80] Klionsky DJ, Emr SD. Autophagy as a regulated pathway of cellular degradation. Science. 2000;290:1717–21. 11099404 10.1126/science.290.5497.1717 PMC2732363

[81] Levine B, Mizushima N, Virgin HW. Autophagy in immunity and inflammation. Nature. 2011;469:323–35. 21248839 10.1038/nature09782 PMC3131688

[82] Dikic I, Elazar Z. Mechanism and medical implications of mammalian autophagy. Nat Rev Mol Cell Biol. 2018;19:349–64. 29618831 10.1038/s41580-018-0003-4

[83] Yu L, Chen Y, Tooze SA. Autophagy pathway: cellular and molecular mechanisms. Autophagy. 2017;14:207–15. 28933638 10.1080/15548627.2017.1378838 PMC5902171

[84] Lőrincz P, Juhász G. Autophagosome-lysosome fusion. J Mol Biol. 2020;432:2462–82. 31682838 10.1016/j.jmb.2019.10.028

[85] Liu Y, Shoji-Kawata S, Sumpter RM, Wei Y, Ginet V, Zhang L, et al. Autosis is a Na+,K+-ATPase-regulated form of cell death triggered by autophagy-inducing peptides, starvation, and hypoxia-ischemia. Proc Natl Acad Sci USA. 2013;110:20364–71. 24277826 10.1073/pnas.1319661110 PMC3870705

[86] Stockwell BR, Friedmann Angeli JP, Bayir H, Bush AI, Conrad M, Dixon SJ, et al. Ferroptosis: a regulated cell death nexus linking metabolism, redox biology, and disease. Cell. 2017;171:273–85. 28985560 10.1016/j.cell.2017.09.021 PMC5685180

[87] Chen X, Kang R, Kroemer G, Tang D. Ferroptosis in infection, inflammation, and immunity. J Exp Med. 2021;218:e20210518. 33978684 10.1084/jem.20210518 PMC8126980

[88] Tang D, Chen X, Kang R, Kroemer G. Ferroptosis: molecular mechanisms and health implications. Cell Res. 2021;31:107–25. 33268902 10.1038/s41422-020-00441-1 PMC8026611

[89] Kruszewski M. Labile iron pool: the main determinant of cellular response to oxidative stress. Mutat Res Mol Mech Mutagen. 2003;531:81–92. 10.1016/j.mrfmmm.2003.08.004 14637247

[90] Ganz T, Nemeth E. Iron homeostasis in host defence and inflammation. Nat Rev Immunol. 2015;15:500–10. 26160612 10.1038/nri3863 PMC4801113

[91] Yuan J, Ofengeim D. A guide to cell death pathways. Nat Rev Mol Cell Biol. 2024;25:379–95. 38110635 10.1038/s41580-023-00689-6

[92] Parker JL, Deme JC, Kolokouris D, Kuteyi G, Biggin PC, Lea SM, et al. Molecular basis for redox control by the human cystine/glutamate antiporter system xc. Nat Commun. 2021;12:7147. 34880232 10.1038/s41467-021-27414-1 PMC8654953

[93] Banjac A, Perisic T, Sato H, Seiler A, Bannai S, Weiss N, et al. The cystine/cysteine cycle: a redox cycle regulating susceptibility versus resistance to cell death. Oncogene. 2008;27:1618–28. 17828297 10.1038/sj.onc.1210796

[94] Yang WS, SriRamaratnam R, Welsch ME, Shimada K, Skouta R, Viswanathan VS, et al. Regulation of ferroptotic cancer cell death by GPX4. Cell. 2014;156:317–31. 24439385 10.1016/j.cell.2013.12.010 PMC4076414

[95] Ursini F, Maiorino M. Lipid peroxidation and ferroptosis: The role of GSH and GPx4. Free Radic Biol Med. 2020;152:175–85. 32165281 10.1016/j.freeradbiomed.2020.02.027

[96] Li J, Cao F, Yin HL, Huang ZJ, Lin ZT, Mao N, et al. Ferroptosis: past, present and future. Cell Death Dis. 2020;11:88. 32015325 10.1038/s41419-020-2298-2 PMC6997353

[97] Mohamed Elashiry M, Tian F, Elashiry M, Zeitoun R, Elsayed R, Andrews ML, et al. *Enterococcus faecalis* shifts macrophage polarization toward M1-like phenotype with an altered cytokine profile. J Oral Microbiol. 2021;13:1868152. 33488991 10.1080/20002297.2020.1868152 PMC7801083

[98] Zou J, Shankar N. *Enterococcus faecalis* infection activates phosphatidylinositol 3-kinase signaling to block apoptotic cell death in macrophages. Infect Immun. 2014;82:5132–42. 25267834 10.1128/IAI.02426-14 PMC4249289

[99] Chi D, Lin X, Meng Q, Tan J, Gong Q, Tong Z. Real-time induction of macrophage apoptosis, pyroptosis, and necroptosis by *Enterococcus faecalis* OG1RF and two root canal isolated strains. Front Cell Infect Microbiol. 2021;11:720147. 34513732 10.3389/fcimb.2021.720147 PMC8427696

[100] Tian Y, Zhang X, Zhang K, Song Z, Wang R, Huang S, et al. Effect of Enterococcus faecalis lipoteichoic acid on apoptosis in human osteoblast-like cells. J Endod. 2013;39:632–7. 23611381 10.1016/j.joen.2012.12.019

[101] Li Y, Tong Z, Ling J. Effect of the three *Enterococcus faecalis* strains on apoptosis in MC3T3 cells. Oral Dis. 2019;25:309–18. 29729070 10.1111/odi.12883

[102] Li Y, Wen C, Zhong J, Ling J, Jiang Q. *Enterococcus faecalis* OG1RF induces apoptosis in MG63 cells via caspase-3/-8/-9 without activation of caspase-1/GSDMD. Oral Dis. 2022;28:2026–35. 34370363 10.1111/odi.13996

[103] Li Y, Sun S, Wen C, Zhong J, Jiang Q. Effect of *Enterococcus faecalis* OG1RF on human calvarial osteoblast apoptosis. BMC Oral Health. 2022;22:279. 35804353 10.1186/s12903-022-02295-y PMC9264677

[104] Ribeiro-Sobrinho AP, Rabelo FL, Figueiredo CB, Alvarez-Leite JI, Nicoli JR, Uzeda M, et al. Bacteria recovered from dental pulp induce apoptosis of lymph node cells. J Med Microbiol. 2005;54:413–6. 15770029 10.1099/jmm.0.45728-0

[105] Dai X, Ma R, Jiang W, Deng Z, Chen L, Liang Y, et al. *Enterococcus faecalis*-induced macrophage necroptosis promotes refractory apical periodontitis. Microbiol Spectr. 2022;10:e01045–22. 35708336 10.1128/spectrum.01045-22 PMC9431707

[106] Ke X, Lei L, Li H, Li H, Yan F. Manipulation of necroptosis by Porphyromonas gingivalis in periodontitis development. Mol Immunol. 2016;77:8–13. 27449906 10.1016/j.molimm.2016.07.010

[107] Cheng R, Feng Y, Zhang R, Liu W, Lei L, Hu T. The extent of pyroptosis varies in different stages of apical periodontitis. Biochim Biophys Acta Mol Basis Dis. 2018;1864:226–37. 29066283 10.1016/j.bbadis.2017.10.025

[108] Ran S, Huang J, Liu B, Gu S, Jiang W, Liang J. *Enterococcus faecalis* activates NLRP3 inflammasomes leading to increased interleukin-1 beta secretion and pyroptosis of THP-1 macrophages. Micro Pathog. 2021;154:104761. 10.1016/j.micpath.2021.104761 33524566

[109] Okano T, Ashida H, Suzuki S, Shoji M, Nakayama K, Suzuki T. Porphyromonas gingivalis triggers NLRP3-mediated inflammasome activation in macrophages in a bacterial gingipains-independent manner. Eur J Immunol. 2018;48:1965–74. 30280383 10.1002/eji.201847658

[110] Park E, Na HS, Song YR, Shin SY, Kim YM, Chung J. Activation of NLRP3 and AIM2 inflammasomes by *Porphyromonas gingivalis* infection. Infect Immun. 2014;82:112–23. 10.1128/IAI.00862-13 PMC3911849 24126516

[111] Huynh NCN, Everts V, Pavasant P, Ampornaramveth RS. Interleukin-1β induces human cementoblasts to support osteoclastogenesis. Int J Oral Sci. 2017;9:e5. 29235551 10.1038/ijos.2017.45 PMC5729550

[112] Pyrillou K, Burzynski LC, Clarke MCH. Alternative pathways of IL-1 activation, and its role in health and disease. Front Immunol. 2020;11:613170. 33391283 10.3389/fimmu.2020.613170 PMC7775495

[113] Wang K, Liu J, Yue J, Zhou L, Mao H, Li J, et al. Nlrp3 inflammasome drives regulatory T cell depletion to accelerate periapical bone erosion. Int Endod J. 2024;57:1110–23. 38441141 10.1111/iej.14062

[114] Liu H, Liu Y, Fan W, Fan B. *Fusobacterium nucleatum* triggers proinflammatory cell death via Z-DNA binding protein 1 in apical periodontitis. Cell Commun Signal. 2022;20:196. 36539813 10.1186/s12964-022-01005-z PMC9764563

[115] Li J, Yue Y, Chan W, Wei W, Liu X, Wang M, et al. RGS10 negatively regulates apical periodontitis via TFEB-mediated autophagy in BABL/c mice model and in vitro. Int Endod J. 2023;56:854–68. 37092953 10.1111/iej.13924

[116] Bolat N, Erzurumlu Y, Aşcı H, Özmen Ö, Üreyen Kaya B. Selenium ameliorates inflammation by decreasing autophagic flux and mitogen-activated protein kinase signalling on experimentally induced rat periapical lesions. Int Endod J. 2023;56:227–44. 36314140 10.1111/iej.13861

[117] Eltzschig HK, Carmeliet P. Hypoxia and inflammation. N Engl J Med. 2011;364:656–65. 21323543 10.1056/NEJMra0910283 PMC3930928

[118] Huang HY, Wang WC, Lin PY, Huang CP, Chen CY, Chen YK. The roles of autophagy and hypoxia in human inflammatory periapical lesions. Int Endod J. 2018;51:e125–45. 28439929 10.1111/iej.12782

[119] Yang M, Shen Z, Zhang X, Song Z, Zhang Y, Lin Z, et al. Ferroptosis of macrophages facilitates bone loss in apical periodontitis via NRF2/FSP1/ROS pathway. Free Radic Biol Med. 2023;208:334–47. 37619958 10.1016/j.freeradbiomed.2023.08.020

[120] Tang Y, Su S, Yu R, Liao C, Dong Z, Jia C, et al. Unraveling ferroptosis in osteogenic lineages: implications for dysregulated bone remodeling during periodontitis progression. Cell Death Discov. 2024;10:195. 38670955 10.1038/s41420-024-01969-6 PMC11053120

[121] Conti LC, Segura-Egea JJ, Cardoso CBM, Benetti F, Azuma MM, Oliveira PHC, et al. Relationship between apical periodontitis and atherosclerosis in rats: lipid profile and histological study. Int Endod J. 2020;53:1387–97. 32573791 10.1111/iej.13350

[122] Wu Y, Sun H, Yang B, Liu X, Wang J. 5-Lipoxygenase knockout aggravated apical periodontitis in a murine model. J Dent Res. 2018;97:442–50. 29125911 10.1177/0022034517741261

[123] Azuma MM, Gomes-Filho JE, Ervolino E, Cardoso CDBM, Pipa CB, Kawai T, et al. Omega-3 fatty acids reduce inflammation in rat apical periodontitis. J Endod. 2018;44:604–8. 29397217 10.1016/j.joen.2017.12.008

[124] Wang H-W, Kok S-H, Yang C-N, Hong C-Y, Chi C-W, Chen M-H, et al. Blockade of fatty acid signalling inhibits lipopolysaccharide-induced macrophage recruitment and progression of apical periodontitis. Int Endod J. 2021;54:902–15. 33369764 10.1111/iej.13468

[125] Lin LM, Huang GTJ, Rosenberg PA. Proliferation of epithelial cell rests, formation of apical cysts, and regression of apical cysts after periapical wound healing. J Endod. 2007;33:908–16. 17878074 10.1016/j.joen.2007.02.006

[126] Lai EHH, Hong CY, Kok SH, Hou KL, Chao LH, Lin LD, et al. Simvastatin alleviates the progression of periapical lesions by modulating autophagy and apoptosis in osteoblasts. J Endod. 2012;38:757–63. 22595108 10.1016/j.joen.2012.02.023

[127] Yang CN, Kok SH, Wang HW, Chang JZC, Lai EHH, Shun CT, et al. Simvastatin alleviates bone resorption in apical periodontitis possibly by inhibition of mitophagy-related osteoblast apoptosis. Int Endod J. 2019;52:676–88. 30537112 10.1111/iej.13055

[128] An Y, Liu W, Xue P, Zhang Y, Wang Q, Jin Y. Increased autophagy is required to protect periodontal ligament stem cells from apoptosis in inflammatory microenvironment. J Clin Periodontol. 2016;43:618–25. 26990245 10.1111/jcpe.12549

[129] Montaseri A, Giampietri C, Rossi M, Riccioli A, Del Fattore A, Filippini A. The role of autophagy in osteoclast differentiation and bone resorption function. Biomolecules. 2020;10:1398. 33008140 10.3390/biom10101398 PMC7601508

[130] Wu Z, Li M, Ren X, Zhang R, He J, Cheng L, et al. Double-edged sword effect of pyroptosis: the role of caspase-1/-4/-5/-11 in different levels of apical periodontitis. Biomolecules. 2022;12:1660. 36359010 10.3390/biom12111660 PMC9687662

[131] Sartoretto S, Gemini-Piperni S, da Silva RA, Calasans MD, Rucci N, Pires Dos Santos TM, et al. Apoptosis-associated speck-like protein containing a caspase-1 recruitment domain (ASC) contributes to osteoblast differentiation and osteogenesis. J Cell Physiol. 2019;234:4140–53. 30171612 10.1002/jcp.27226

[132] Liu J, Wang J, Ren J, Yang Q, Zhan W, Wang M, et al. Inhibition of receptor-interacting protein kinase-3 in the necroptosis pathway attenuates inflammatory bone loss in experimental apical periodontitis in Balb/c mice. Int Endod J. 2021;54:1538–47. 33896018 10.1111/iej.13534

[133] Jorgensen I, Miao EA. Pyroptotic cell death defends against intracellular pathogens. Immunol Rev. 2015;265:130–42. 25879289 10.1111/imr.12287 PMC4400865

[134] Chen Y, Yang Q, Lv C, Chen Y, Zhao W, Li W, et al. NLRP3 regulates alveolar bone loss in ligature-induced periodontitis by promoting osteoclastic differentiation. Cell Prolif. 2021;54:e12973. 33382502 10.1111/cpr.12973 PMC7849172

[135] Lu WL, Zhang L, Song DZ, Yi XW, Xu WZ, Ye L, et al. NLRP6 suppresses the inflammatory response of human periodontal ligament cells by inhibiting NF-κB and ERK signal pathways. Int Endod J. 2019;52:999–1009. 30712265 10.1111/iej.13091

[136] Zamani P, Oskuee RK, Atkin SL, Navashenaq JG, Sahebkar A. MicroRNAs as important regulators of the NLRP3 inflammasome. Prog Biophys Mol Biol. 2020;150:50–61. 31100298 10.1016/j.pbiomolbio.2019.05.004

[137] Wang D, Sun S, Xue Y, Qiu J, Ye T, Zhang R, et al. MicroRNA-223 negatively regulates LPS-induced inflammatory responses by targeting NLRP3 in human dental pulp fibroblasts. Int Endod J. 2021;54:241–54. 32966618 10.1111/iej.13413

[138] Li C, Yin W, Yu N, Zhang D, Zhao H, Liu J, et al. miR-155 promotes macrophage pyroptosis induced by *Porphyromonas gingivalis* through regulating the NLRP3 inflammasome. Oral Dis. 2019;25:2030–9. 31529565 10.1111/odi.13198

[139] Guan X, Zhao R, Wang Y, Li W, Pan L, Yang Y, et al. Ginsenoside Rb1 ameliorates apical periodontitis via suppressing macrophage pyroptosis. Oral Dis. 2025;31:541–54. 39155466 10.1111/odi.15103

[140] Ou M, Jiang Y, Ji Y, Zhou Q, Du Z, Zhu H, et al. Role and mechanism of ferroptosis in neurological diseases. Mol Metab. 2022;61:101502. 35447365 10.1016/j.molmet.2022.101502 PMC9170779

[141] Wu W, Li G, Dong S, Huihan Chu C, Ma S, Zhang Z, et al. Bomidin attenuates inflammation of periodontal ligament stem cells and periodontitis in mice via inhibiting ferroptosis. Int Immunopharmacol. 2024;127:111423. 38141410 10.1016/j.intimp.2023.111423

[142] Qiao S, Li B, Cai Q, Li Z, Yin Z, He J, et al. Involvement of ferroptosis in *Porphyromonas gingivalis* lipopolysaccharide-stimulated periodontitis in vitro and in vivo. Oral Dis. 2023;29:3571–82. 35765229 10.1111/odi.14292

[143] Wang Y, Li W, Mu W, Seyam A, Guan Y, Tang Y, et al. Identification of JNK-JUN-NCOA axis as a therapeutic target for macrophage ferroptosis in chronic apical periodontitis. Int J Med Sci. 2025;22:53–70. 39744165 10.7150/ijms.102741 PMC11659826

[144] Liu T, Wang L, Liang P, Wang X, Liu Y, Cai J, et al. USP19 suppresses inflammation and promotes M2-like macrophage polarization by manipulating NLRP3 function via autophagy. Cell Mol Immunol. 2021;18:2431–42. 33097834 10.1038/s41423-020-00567-7 PMC8484569

[145] Park OJ, Kim AR, So YJ, Im J, Ji HJ, Ahn KB, et al. Induction of apoptotic cell death by oral streptococci in human periodontal ligament cells. Front Microbiol. 2021;12:738047. 34721337 10.3389/fmicb.2021.738047 PMC8551966

[146] Dai X, Deng Z, Liang Y, Chen L, Jiang W, Zhao W. *Enterococcus faecalis* induces necroptosis in human osteoblastic MG63 cells through the RIPK3/MLKL signalling pathway. Int Endod J. 2020;53:1204–15. 32379949 10.1111/iej.13323

[147] Geng F, Liu J, Yin C, Zhang S, Pan Y, Sun H. *Porphyromonas gingivalis* lipopolysaccharide induced RIPK3/MLKL-mediated necroptosis of oral epithelial cells and the further regulation in macrophage activation. J Oral Microbiol. 2022;14:2041790. 35251521 10.1080/20002297.2022.2041790 PMC8890547

[148] Huang C, Zhang C, Yang P, Chao R, Yue Z, Li C, et al. Eldecalcitol inhibits LPS-induced NLRP3 inflammasome-dependent pyroptosis in human gingival fibroblasts by activating the Nrf2/HO-1 signaling pathway. Drug Des Devel Ther. 2020;ume 14:4901–13. 10.2147/DDDT.S269223 PMC7671541 33223823

[149] Lian D, Dai L, Xie Z, Zhou X, Liu X, Zhang Y, et al. Periodontal ligament fibroblasts migration injury via ROS/TXNIP/Nlrp3 inflammasome pathway with *Porphyromonas gingivalis* lipopolysaccharide. Mol Immunol. 2018;103:209–19. 30312877 10.1016/j.molimm.2018.10.001

[150] Lin D, Gao Y, Zhao L, Chen Y, An S, Peng Z. *Enterococcus faecalis* lipoteichoic acid regulates macrophages autophagy via PI3K/Akt/mTOR pathway. Biochem Biophys Res Commun. 2018;498:1028–36. 29551680 10.1016/j.bbrc.2018.03.109

