# Supplementary material for: Dual role mechanisms of regulated cell death in apical periodontitis: from pathogenic destruction to therapeutic potential
Source: Cell Death Discov. 2025 Aug 15;11:386. doi: 10.1038/s41420-025-02686-4 (PMC12356886; doi:10.1038/s41420-025-02686-4)
Supplement: Supplementary file 1 — Supplementary Table 1 [file 41420_2025_2686_MOESM1_ESM.docx]

Supplementary Table 1. Detailed molecular annotations for all signaling components.

| **Pathway** | **Abbreviation** | **Full Name** | **Function** |
| --- | --- | --- | --- |
| A：Apoptosis | Active-CASP3,6,7 | Activated caspases-3/-6/-7 | Executioner caspases that cleave cellular substrates to dismantle cells |
|  | Active-CASP8 | Activated caspases-8 | Initiator caspase in extrinsic pathway |
|  | Active-CASP9 | Activated caspases-9 | Initiator caspase in intrinsic pathway |
|  | APAF-1 | Apoptotic protease-activating factor 1 | Forms heptameric "apoptosome" complex activating caspase-9 |
|  | Bak | BCL2 antagonist | Forms mitochondrial pores with Bax to release cytochrome c |
|  | Bcl-2 | B-cell lymphoma 2 | Anti-apoptotic protein that sequesters Bax/Bak |
|  | Cyt C | Cytochrome c | Mitochondrial protein released during MOMP, activates APAF-1 |
|  | DISC | Death-inducing signaling complex | Plasma membrane complex initiating extrinsic apoptosis |
|  | FADD | FAS-associated death domain | Adaptor protein bridging death receptors and caspase-8 |
|  | MOMP | Mitochondrial outer membrane permeabilization | Critical event in intrinsic apoptosis mediated by Bax/Bak pores |
|  | Pro-CASP3, 6, 7 | Pro caspases-3/-6/-7 | Inactive zymogens of executioner caspases |
|  | Pro-CASP8 | Pro caspases-8 | Inactive precursor of caspase-8 |
|  | Pro-CASP9 | Pro caspases-9 | Inactive precursor of caspase-9 |
|  | tBid | Truncated BH3-interacting domain death agonist | Activated by caspase-8, translocates to mitochondria to activate Bax/Bak |
|  | TRADD | TNF receptor 1-associated death domain protein | Adaptor protein recruiting FADD/RIPK1 in TNFR1 signaling |
| B: Necroptosis | Active-CASP8 | Activated caspase-8 | Inhibits necroptosis by cleaving RIPK1/RIPK3 |
|  | CASP8 | Caspase-8 | Dual-function protease: apoptosis activation/necroptosis suppression |
|  | cIAP1/2 | inhibitor of apoptosis protein 1/2 | E3 ubiquitin ligases that suppress RIPK1 activation |
|  | PAMPs | Pathogen-associated molecular patterns | Microbial components triggering PRR ctivation |
|  | FADD | FAS-associated death domain | Component of necrosome complex in caspase-8 deficient conditions |
|  | MLKL | Mixed lineage kinase domain-like | Pseudokinase forming plasma membrane pores upon RIPK3 phosphorylation |
|  | RIPK1 | Receptor-interacting protein kinase 1 | Scaffold kinase activating RIPK3 in necrosome complex |
|  | RIPK3 | Receptor-interacting protein kinase 3 | Key kinase phosphorylating MLKL to execute necroptosis, activated downstream of RIPK1 or pathogen sensors |
|  | TRADD | TNF receptor 1-associated death domain protein | TNFR1 adaptor recruiting RIPK1 |
|  | TRAF2/5 | TNF receptor-associated factor 2/5 | E3 ubiquitin ligases regulating RIPK1 ubiquitination status |
| C: Pyroptosis | Active-CASP1 | Activated caspase-1 | Processes GSDMD and pro-IL-1β/pro-IL-18 in canonical pathway |
|  | Active-CASP4,5,11 | Activated caspases-4/-5/-11 | Inflammatory caspases cleaving GSDMD in non-canonical pathway |
|  | GSDMD | Gasdermin D | Pore-forming executor of pyroptosis (N-terminal domain) |
|  | IL-18 | Interleukin-18 | Proinflammatory cytokine matured by caspase-1 |
|  | IL-1β | Interleukin-1 beta | Pyroptosis-associated cytokine activated by inflammasomes |
|  | N-GSDMD | N-terminal gasdermin D | Active pore-forming fragment released by caspase cleavage |
|  | PAMPs | Pathogen-associated molecular patterns | Ligands for NLRP3/other inflammasome sensors |
|  | Pro-CASP4,5,11 | Pro caspases-4/-5/-11 | Inactive precursors of non-canonical inflammasome caspases |
|  | Pro-IL-18 | Pro-interleukin-18 | Inactive precursor cleaved by caspase-1 |
|  | Pro-IL-1β | Pro-interleukin-1 beta | Inactive precursor processed by caspase-1 |
| D: PANoptosis | AIM2 | Absent in melanoma 2 | Cytosolic DNA sensor activating inflammasome |
|  | ASC | Apoptosis-associated speck-like protein | Adaptor protein polymerizing to form inflammasome specks |
|  | CASP1 | Caspase-1 | Executor of pyroptosis component in PANoptosome |
|  | CASP8 | Caspase-8 | Apoptosis initiator with cross-talk roles in PANoptosis |
|  | FADD | FAS-associated death domain | Platform for caspase-8 activation in PANoptosome |
|  | MLKL | Mixed lineage kinase domain-like | Necroptosis executor integrated in PANoptosis pathways |
|  | NLRC5 | NLR family CARD domain containing 5 | Regulator of inflammatory cell death cross-talk |
|  | NLRP12 | NLR family pyrin domain containing 12 | Sensor for bacterial metabolites in PANoptosis |
|  | NLRP3 | NLR family pyrin domain containing 3 | Central inflammasome sensor in PANoptosis |
|  | Pyrin | MEFV protein | Sensor for pathogen modifications (e.g., Rho GTPase inactivation) |
|  | RIPK1 | Receptor-interacting protein kinase 1 | Kinase scaffold bridging apoptosis/necroptosis pathways |
|  | RIPK3 | Receptor-interacting protein kinase 3 | MLKL activator in necroptosis arm of PANoptosis |
|  | ZBP1 | Z-DNA binding protein 1 | Master sensor of viral infection triggering PANoptosis |
| E: Autophagy-dependent cell death | AMBRA1 | Autophagy and beclin 1 regulator 1 | Activator of Beclin-1-VPS34 complex during nucleation |
|  | ATG101 | Autophagy related 101 | Stabilizer of ULK1 complex under nutrient stress |
|  | ATG12 | Autophagy related 12 | Conjugated to ATG5 forming E3-like enzyme for LC3 lipidation |
|  | ATG13 | Autophagy related 13 | ULK1-binding protein regulating autophagosome initiation |
|  | ATG14 | Autophagy related 14 | PI3KC3 complex component specifying autophagic membranes |
|  | Beclin1 | BECN1 (coiled-coil moesin-like BCL2-interacting protein) | Scaffold for PI3KC3 complex assembly |
|  | FIP200 | RB1-inducible coiled-coil protein 1 | ULK1-binding partner in initiation complex |
|  | LC3 | Microtubule associated protein 1 light chain 3 | Phosphatidylethanolamine-conjugated phagophore marker |
| F: Ferroptosis | GPX4 | Glutathione peroxidase 4 | Key antioxidant enzyme reducing lipid peroxides |
|  | GSH | Glutathione | Tripeptide cofactor required for GPX4 activity |
|  | PUFA-PL-OH | Reduced phospholipid alcohols | Non-toxic end-products of lipid peroxide reduction |
|  | PUFA-PL-OOH | Phospholipid hydroperoxides | Oxidized PUFAs triggering membrane permeabilization |
|  | PUFAs | Polyunsaturated fatty acids | Oxidation-prone lipids |
|  | ROS | Reactive oxygen species | Drivers of lipid peroxidation cascade |
|  | System Xc- | Cystine/glutamate antiporter | Transporter importing cystine for glutathione synthesis |
